# Maintenance of autoantibody production in pristane-induced murine lupus

**DOI:** 10.1186/s13075-015-0886-9

**Published:** 2015-12-30

**Authors:** Shuhong Han, Haoyang Zhuang, Yuan Xu, Pui Lee, Yi Li, Joseph C. Wilson, Osvaldo Vidal, Hong Seok Choi, Yu Sun, Li-Jun Yang, Westley H. Reeves

**Affiliations:** Department of Medicine, Division of Rheumatology & Clinical Immunology, University of Florida, 1600 Archer Road, Gainesville, FL 32610-0275 USA; College of Pharmacy, University of Florida, Student Service Center, HPNP Complex, PO Box 100495, Gainesville, FL 32610-0495 USA; Department of Molecular genetics and Microbiology, University of Florida, PO Box 100221, Gainesville, FL 32610-0221 USA; Department of Pathology and Laboratory Medicine, University of Florida, 1395 Center Dr., Gainesville, FL 32610-0495 USA; Current Address: Boston Children’s Hospital, 300 Longwood Ave, Boston, MA 02115 USA; Current Address: Qilu Hospital of Shandong University, Jinan, 250012 PR China

**Keywords:** Pristane, Lupus, Memory B cells, TLR7, Anti-RNP/Sm autoantibody

## Abstract

**Background:**

Pristane-treated mice chronically produce high levels of anti-ribonucleoprotein/Smith (anti-Sm/RNP) and other lupus autoantibodies. The present study addressed how these autoantibody levels are maintained over time.

**Methods:**

Lupus was induced in BALB/c mice using pristane. Naïve B cells, switched memory B cells, switched plasmablasts, and plasma cells were flow-sorted and total IgG and anti-U1A (RNP) autoantibodies were determined with ELISA.

**Results:**

B cells with a switched “memory-like” (CD19^+^CD138^−^IgM^−^IgD^−^) (sMB) phenotype were increased in pristane-treated mice and expressed higher levels of Toll like receptor 7 (*Tlr7*) than cells with this phenotype from untreated mice. Flow-sorted sMB cells from pristane-treated mice did not secrete IgG spontaneously, but were hyper-responsive to both synthetic (R848) and natural (apoptotic cells) TLR7 ligands, resulting in increased IgG production *in vitro*. The flow-sorted sMB cells also could be driven by R848 to produce IgG anti-U1A autoantibodies. Production of IgG was strongly inhibited by both JSH-23 and SB203580, suggesting that the canonical NFκB and p38 MAPK pathways, respectively, contribute to the TLR7 ligand hyper-responsiveness of sMB from pristane-treated mice.

**Conclusions:**

The switched memory B cell subset from pristane-treated mice is expanded and shows an increased propensity to undergo terminal (plasma cell) differentiation in response to synthetic and natural TLR7 ligands. The data suggest that the decreased clearance of apoptotic cells characteristic of pristane-treated mice might help maintain high serum levels of anti-RNP/Sm autoantibodies.

**Electronic supplementary material:**

The online version of this article (doi:10.1186/s13075-015-0886-9) contains supplementary material, which is available to authorized users.

## Background

Systemic lupus erythematosus (SLE) is characterized by B cell hyperactivity and autoantibody production against nucleic acid-associated proteins, such as anti-Smith/ribonucleoprotein (anti-Sm/RNP), anti-DNA, and others [1]. Selectivity for these autoantigens may relate to their associated nucleic acids, which are ligands for toll-like receptors (TLR) [2–4]. TLR7 is involved in innate immunity to pathogen-associated single-stranded RNA [5] and also recognizes host RNA sequences including U1 RNA, the RNA component of U1 small nuclear ribonucleoproteins (snRNPs) recognized by anti-Sm/RNP autoantibodies [2]. Accumulating evidence implicates TLR7-driven type I interferon (IFN-I) production as a key mediator of murine lupus [6]. In experimental lupus induced by 2,6,10,14-tetramethylpentadecane (TMPD, pristane), autoantibody production and nephritis are abolished in mice lacking either TLR7 or the IFN-I receptor [7]. TLR7 is IFN-I inducible [8], suggesting that increased TLR7 expression may contribute to B cell hyperactivity and autoantibody production in SLE [9]. RNA-protein complexes can activate B cells through dual engagement of the B cell receptor and TLR7 [4]. For autoantibody production, TLR7 could be involved in the initial breach of tolerance, the maintenance of serological memory once tolerance has been broken, or the survival of autoreactive B cells [10, 11]. Various B cell subsets implicated in autoantibody production, including follicular, marginal zone, and B-1a/B-1b B cells, respond differently to TLR7 signaling [12–14]. Moreover, TLR7 signaling could enhance autoantibody production by plasmablasts (PB) or plasma cells (PC) or could promote the differentiation of memory B cells (MB) into antibody-secreting PB/PC.

We previously reported that the bone marrow (BM) of mice with pristane-induced lupus contains autoreactive B cells specific for the RNP autoantigen U1A [15]. These cells have some characteristics of switched memory B cells (sMB), including the absence of surface IgM and lack of spontaneous IgG secretion. Upon stimulation with the TLR4 ligand lipopolysaccharide (LPS), they differentiate into anti-U1A antibody-secreting cells. More recently, we found that BM in SLE patients and in pristane-treated mice contains numerous dead cells, which might stimulate local TNFα production by engaging TLR7 [16]. It is not known whether autologous TLR7 ligands derived from dead cells can drive terminal differentiation of anti-U1A MB.

In the present study, we found that sMB from pristane-treated mice are hyper-responsive to the synthetic TLR7 ligand (R848), probably resulting from increased TLR7 expression due to IFN-I production. We show that in pristane-treated mice, an expanded population of sMB can become IgG-secreting PB in response to R848 or apoptotic cells, raising the possibility that the accumulation of dead cells in lupus tissues may help maintain autoantibody production by driving terminal B cell differentiation.

## Methods

### Mice

Mice were bred and maintained under specific pathogen-free conditions at the University of Florida Animal Facility. BALB/cByJ mice were from Jackson Laboratory (Bar Harbor, ME, USA). BALB/c TLR7^−/−^ mice were provided originally by Dr. Shizuo Akira and bred in our facility. To induce lupus, 0.5 ml of pristane was administered by intraperitoneal (i.p.) injection. Controls were treated with PBS. Spleen was harvested 12 months after treatment. There were two to six mice per group and experiments were repeated separately at least three times. These studies were approved by the Institutional Animal Care and Use Committee, University of Florida.

### Reagents and cell lines

R848 (TLR7 ligand) and CpG-B ODN 1826 (murine TLR9 ligand) were purchased from Invivogen (San Diego, CA, USA). Lipopolysaccharide (LPS, TLR4 ligand from *Salmonella Minnesota*) was from Sigma-Aldrich(St. Louis, MO). Recombinant murine BAFF was from Biolegend (San Diego, CA). ODN2088 and ODN20958 were from Miltenyi Biotec GmbH (Bergisch Gladbach, Germany). JSH23 (4-methyl-*N*1-(3-phenyl-propyl)-benzene-1,2-diamine, Sigma-Aldrich), SB203580 (4-(4-fluorophenyl)-2-(4-methylsulfinylphenyl)-5-(4-pyridyl)-1H-imidazole) (Sigma-Aldrich). Amino-actinomycin D (7-AAD) was obtained from BD Bioscience (San Jose, CA, USA). The non-secreting mouse myeloma cell line SP2/0 was obtained from the American Type Culture Collection (ATCC, Manassas, VA, USA). The murine anti-U1-70 K (RNP) hybridoma cell line 2.73 was a gift of Dr. Sally Hoch [17]. Additional hybridomas included 172.4 (murine anti-CD100, ATCC), 11B11 (rat IgG1 anti-mouse IL-4, ATCC), Pab101 (murine IgG2a anti-SV40 large T antigen, ATCC), 111 (murine IgG1 anti-Ku), and 162 (murine IgG2a anti-Ku).

### Reverse transcriptase-polymerase chain reaction (RT-PCR)

Total RNA was isolated with TRIZOL reagent (Sigma-Aldrich). cDNA was synthesized using the Superscript II First-Strand Synthesis kit (Invitrogen) according to the manufacturer’s protocol. Real-time PCR was carried out as described [16]. Primer sequences were as follows: TLR1 forward, CATTCCTGAGGTCCCTGCTA, reverse, GATGCACAGCTCCTTGGTTT; TLR2 forward, CACCAAGATCCAGAAGAGCC and reverse TAGGGGCTTCACTTCTCTGC; TLR3 forward, ACAAAAGTCCCCCAAAGGAG and reverse TGCAGTCTTTCCAGAGGGAT; TLR4 forward, TGTCATCAGGGACTTTGCTG and reverse GGACTCTGATCATGGCACTG; TLR5 forward, GCACAGAAAGCATGAAGCTG and reverse GCCTCTAAGGGCTCTCACCT; TLR6 forward, GAGGCTATCCCAGAGGGACT and reverse GAAGGCTTTTCTGGATTTTTCA; TLR7 forward, TGAGGGACTTCCCACTAACA and reverse TTGGCTTTGGACCCCAGTAG; TLR8 forward, CGCTTTATGGAAGATGGCAC and reverse TGGATGTTAAGAGAGAAACAAACG; TLR9 forward, GGCTTCAGCTCACAGGGTAG and reverse GAATCCTCCATCTCCCAACA; CIITA forward, TCAAGCACATTGGAGCAGAG and reverse AGGTCCTAGAGGTGGGCACT; UNC93B1 ATAGATGCCCACAGCCAAGA and reverse TGCTGATGGGTATCAACGTG; 18 s forward, CGGCTACCACATCCAAGGAA and reverse GCTGGAATTACCGCGGCT.

### Flow cytometry

Monoclonal antibodies (mAbs) against mouse CD19 (clone 6D5, Pacific blue), CD138 (clone 281–2, APC), IgD (clone 11-26c.2a, PE) were from Biolegend(San Diego, CA). The mAbs against mouse IgD (clone 11-26c.2a, FITC), I-A/I-E (M5/114.15.2, PE), B220 (clone RA3-6B2, PerCP-Cy5.5) and IgM (clone II/41, PerCP-cy5.5) were from BD Bioscience. Cells were analyzed on LSRII flow cytometer (BD Bioscience). Data were analyzed using FlowJo software (Tree Star Inc., Ashland, OR, USA).

### Isolation of B cells and B cell subsets

Total B cells were positively selected from splenocytes using a B cell magnetic bead isolation kit (Miltenyi Biotec, Auburn, CA, USA). Purity was >95 %. Murine B cell subpopulations were sorted with FACSaria (BD Biosciences). The following B cell subpopulations were sorted: naïve B cells (NB, CD19^+^CD138^−^IgM^+^IgD^+^); switched “memory-like” B cells (sMB, CD19^+^CD138^−^IgM^−^IgD^−^); switched plasmablasts (sPB, CD19^+^CD138^+^IgM^−^IgD^−^); and plasma cells (PC, CD19^+^CD138^++^IgM^−^IgD^−^). Purity was generally >95 %. The sorted cells were cultured or lysed with Trizol for harvesting RNA.

### B cell culture and ELISA

Sorted B cells were seeded at 2.0 × 10^6^ cells/ml with various stimulation conditions: R848 (1 μg/ml), LPS (1 μg/ml) and CpG 1826(10 μg/ml). In some experiments, ODN 2088 and ODN 20958 were added (0.1 μM) with R848. Ten days later, culture supernatants were harvested and tested for antibody production by ELISA. To test the role of NFκB signaling and p38 mitogen-activated protein kinase (MAPK) in IgG production, JSH-23 (NF-kB inhibitor)[18] and SB203580 (p38 MAPK inhibitor) [19] was added. B cells were treated with inhibitors for 1 h before adding R848.

In some experiments, positively selected B cells (2.0 × 10^6^/ml) were co-cultured with apoptotic BW5147 (mouse thymoma cell line, from ATCC) for 10 d at a 1:1 ratio before testing the supernatants by ELISA. BW5147 cells were heat-shocked at 45 °C for 10 minutes and then cultured 4 h before harvesting the apoptotic cells as described [20].

Isolated B cell subsets (5 × 10^5^ cells/ml) were cultured for 10 days in RPMI-1640 supplemented with 10 % fetal bovine serum with/without R848 (1 μg/ml). Total IgG/IgM or IgG anti-U1A autoantibodies in supernatants were quantified by ELISA as described [21].

### Responses of murine hybridomas to TLR7 stimulation

SP2/0 and hybridoma cells were seeded at 4 × 10^4^/ml and stimulated for 3 days with R848 (1 μg/ml), LPS (1 μg/ml), or CpG1826 (10 μg/ml) before harvesting culture supernatants for immunoglobulin ELISA. To assess the effect of TLR ligands on proliferation, hybridoma cells were loaded with carboxyfluorescein diacetate succinimidyl ester (CFSE, CellTrace CFSE cell proliferation kit, Molecular Probes, Eugene, OR, USA) according to the manufacturer’s protocol. CFSE-loaded cells were cultured for 3 days in the presence of R848, LPS and CpG1826. CFSE fluorescence intensity was evaluated by flow cytometry as above.

### Statistical analysis

Data are presented as mean ± SD. For normally distributed data, comparisons between mean values were performed by the unpaired or paired two-tailed Student *t* test using GraphPad (San Diego, CA, USA) Prism version-5 software. Comparisons for non-normally distributed data were made using the *t* test; *p* <0.05 was considered significant.

## Results

In pristane-induced lupus, murine B cells that do not spontaneously secrete anti-U1A (RNP) autoantibodies can be driven to produce autoantibodies by culturing with LPS [15]. We examined the B cell subset(s) that develop into autoantibody-secreting cells and investigated whether other TLR ligands also can promote terminal differentiation of these cells.

### Pristane treatment alters TLR7 responsiveness

**Fig. 1:**
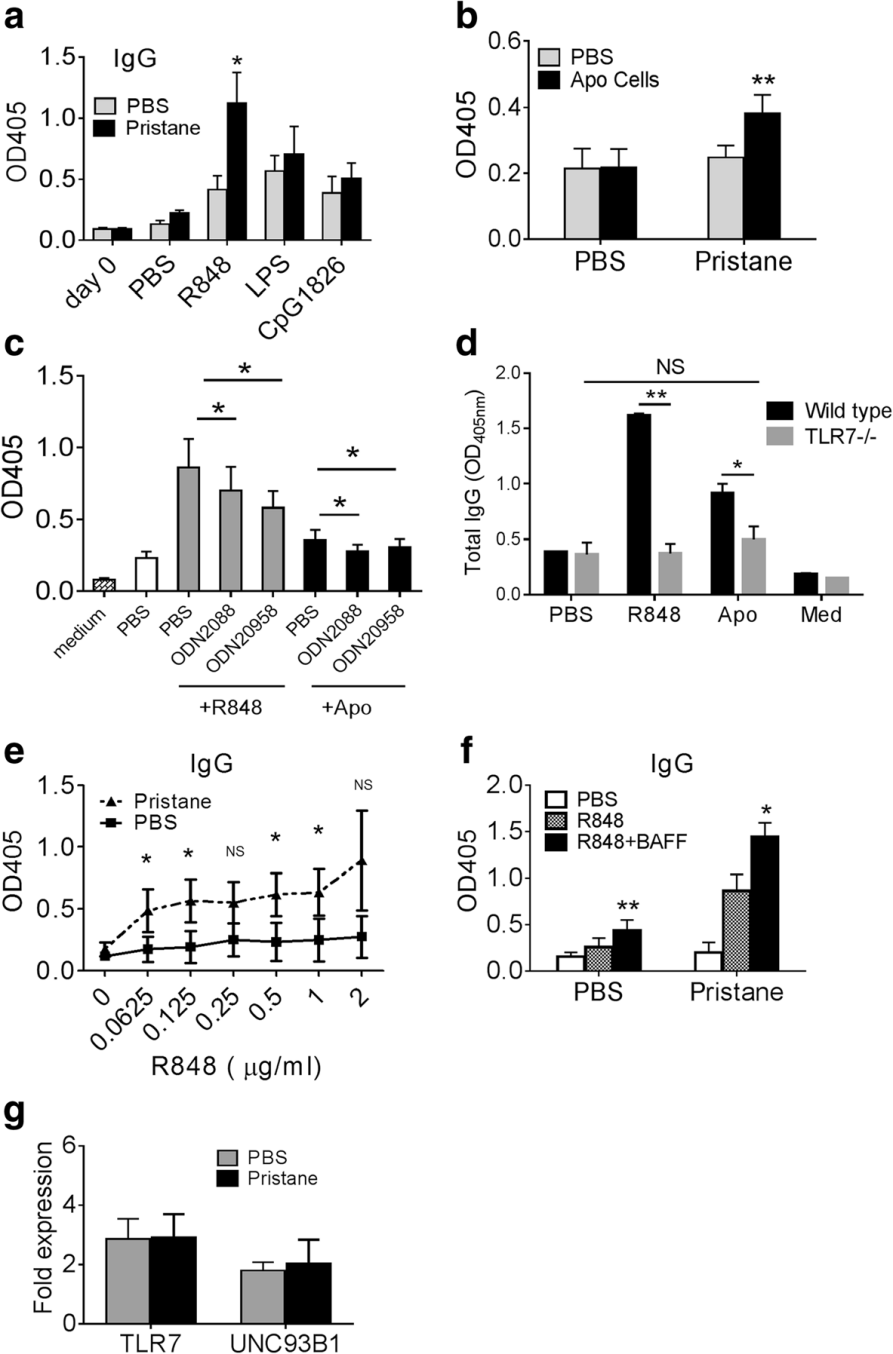
Splenic CD19^+^ B cells from pristane-treated mice are hyper-responsive to synthetic toll-like receptor (*TLR*)7 ligand and apoptotic cells. **a** Purified (magnetic beads) splenic CD19^+^ B cells from pristane-treated (one year) or PBS treated mice were stimulated by PBS or TLR ligands R848 (TLR7), lipopolysaccharide (*LPS*) (TLR4) and CpG1826 (TLR9) for 10 days. IgG was measured in culture supernatants (ELISA). **b** Splenic CD19^+^ B cells were stimulated by PBS or apoptotic BW5147 cells (*Apo cells*) (Apo cells: B cells = 1:1) and IgG was measured 10 days later by ELISA. **c** CD19^+^ B cells were stimulated by R848 or apoptotic cells with addition of ODN 2088 (0.1 μM) or ODN 20958 (0.1 μM), and IgG was measured 10 days later by ELISA. **d** TLR7−/− and wild type mice were treated with pristane for 3 months. Purified splenic CD19^+^ B cells were stimulated with PBS, R848 (1 μg/ml), or apoptotic BW5147 (Apo cells: B cells = 1:1). IgG in culture supernatants was measured 10 days later by ELISA. **e** R848 dose–response curves for CD19^+^ B cells from pristane-treated vs. PBS treated mice. IgG levels were measured by ELISA. **f** CD19^+^ B cells were cultured with R848 with/without the addition of recombinant murine BAFF (5 ng/ml, Biolegend) followed by measurement of IgG in culture supernatants (ELISA). **g** *Tlr7* and *Unc93b1* mRNA expression level (compared to 18S rRNA) in pristane-treated vs. PBS treated splenic CD19^+^ B cells (Q-PCR): *p <0.05; **p <0.01, paired Student *t* test. *BAFF* B cell activating factor

Our previous study showed that TLR7 is necessary for disease development in pristane-induced lupus [20]. To assess the effect of pristane treatment on TLR ligand responsiveness, we cultured positively selected splenic CD19^+^ B cells (>95 % purity) from pristane-treated and PBS-treated BALB/c mice for 10 days with LPS, R848, or CpG1826 and found that IgG production was stimulated by all three TLR ligands (Fig. 1a). However, stimulated IgG levels were substantially higher in culture supernatants from pristane-treated vs. PBS-treated mice, especially in the case of R848. In view of recent evidence that the BM of both SLE patients and pristane-treated mice contains numerous dead cells [16] along with IgG anti-U1A memory-like B cells [15], we asked whether purified B cells from pristane-treated mice could secrete IgG in response to apoptotic cells (Fig. 1b). Splenic B cells from PBS-treated mice produced little IgG when co-cultured with apoptotic BW5147 murine thymoma cells. In contrast, B cells purified from pristane-treated mice increased their IgG production when co-cultured with apoptotic cells (Fig. 1b). We hypothesized that apoptotic cells may provide TLR7 ligands that stimulate B cells from pristane-treated mice. To address this question, TLR7 (ODN 20958) and TLR7/8/9 (ODN2088) inhibitors were added into B cells cultured with R848 or apoptotic BW5147 cells. Both ODN2088 and ODN20958 inhibited apoptotic cell-induced IgG production (Fig. 1c). ODN20958 is a selective TLR7 antagonist, and its inhibition of immunoglobulin secretion suggests TLR7 ligands from apoptotic cells might stimulate B cells to produce IgG. That possibility was supported by looking at TLR7−/− mice (Fig. 1d). As expected, R848 stimulated IgG production by purified B cells from wild type, but not TLR7−/− mice. Apoptotic cells also stimulated IgG production by wild type mice. In contrast, IgG production increased only slightly when TLR7−/− B cells were cultured with apoptotic cells, whereas wild type B cells exhibited a stronger response (Fig. 1d).

Next we examined whether apoptotic cells could stimulate B cells to produce IgG due to an intrinsic hyper-responsiveness to TLR7 stimulation in pristane-treated mice. Consistent with that possibility, IgG production by R848-treated B cells from pristane-treated mice was higher than by B cells from untreated controls (Fig. 1e). Addition of B cell activating factor (BAFF) to the cultures further enhanced IgG production in R848-treated B cells from both pristane-treated and control mice (Fig. 1f). There was little difference in *Tlr7* gene expression in total CD19^+^ B cells from pristane-treated mice vs. untreated controls (Fig. 1g). Likewise, there was little difference in the expression of *Unc93b1* (Fig. 1g), which restricts TLR7-mediated inflammation by biasing endosomal TLR responses in favor of TLR9 [22].

### Pristane treatment alters B cell subsets in spleen

**Fig. 2:**
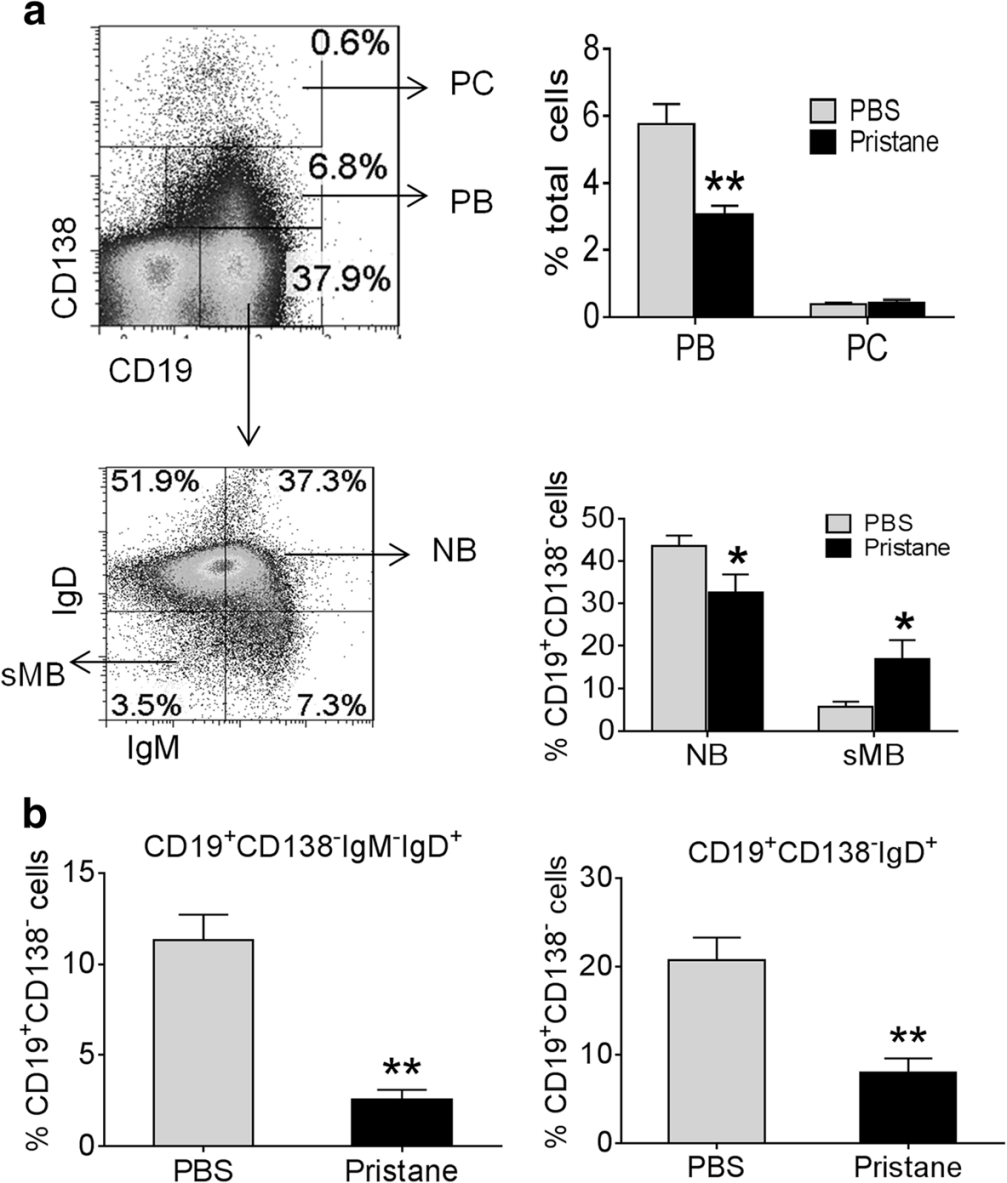
B cell subsets in spleen from pristane-treated vs. PBS treated mice. Spleen cells from pristane-treated (1 year) and age-matched PBS treated mice were stained with anti-CD19, CD138, IgM, and IgD antibodies and analyzed by flow cytometry. **a** Gating strategy for CD19^+^CD138^+^ plasmablasts (*PB*) and CD19^+/−^CD138^++^ plasma cells (*PC*) (*top*) and CD19^+^CD138^−^IgM^+^IgD^+^ naïve B cells (NB), and CD19^+^CD138^−^IgM^−^IgD^−^ switched-memory B cells (*sMB*) (*bottom*) are shown on the left. Percentages of PB and PC in spleen from pristane-treated and untreated mice are on the *top right* and percentages of NB and sMB in CD19^+^CD138^−^ cells are on the *bottom right*. **b** The gating strategy for CD19^+^CD138^−^IgM^−^IgD^+^ and CD19^+^CD138^−^IgD^+^ cells is the same as **a**. The percentage of CD19^+^CD138^−^IgM^−^IgD^+^ and CD19^+^CD138^−^IgD^+^ cells in CD19^+^CD138^−^ cells in spleens from pristane-treated and untreated mice are shown: *p <0.05; **p <0.01, Mann–Whitney test

We next examined the distribution of B cell subsets in pristane-treated vs. control mice by staining for CD19, CD138, IgM and IgD (Fig. 2a). Unexpectedly, total CD19^+^CD138^+^ PB also decreased in pristane-treated spleens (Fig. 2a, top). B cells with an sMB-like phenotype (CD19^+^CD138^−^IgM^−^IgD^−^) were increased in spleens from pristane-treated mice (Fig. 2a, bottom). In contrast, CD19^+^CD138^−^IgM^+^IgD^+^ NB cells were decreased. As there was not a clear separation between the NB population and other cells that were CD19^+^CD138^−^IgM^−^IgD^+^, we also analyzed this population and the combined (CD19^+^CD138^−^IgM^+ or -^IgD^+^) population, and found that cells with these phenotypes were all decreased in pristane-treated mice (Fig. 2b).

### Pristane treatment increases TLR7 expression in sMB and responsiveness to TLR7 ligand

**Fig. 3:**
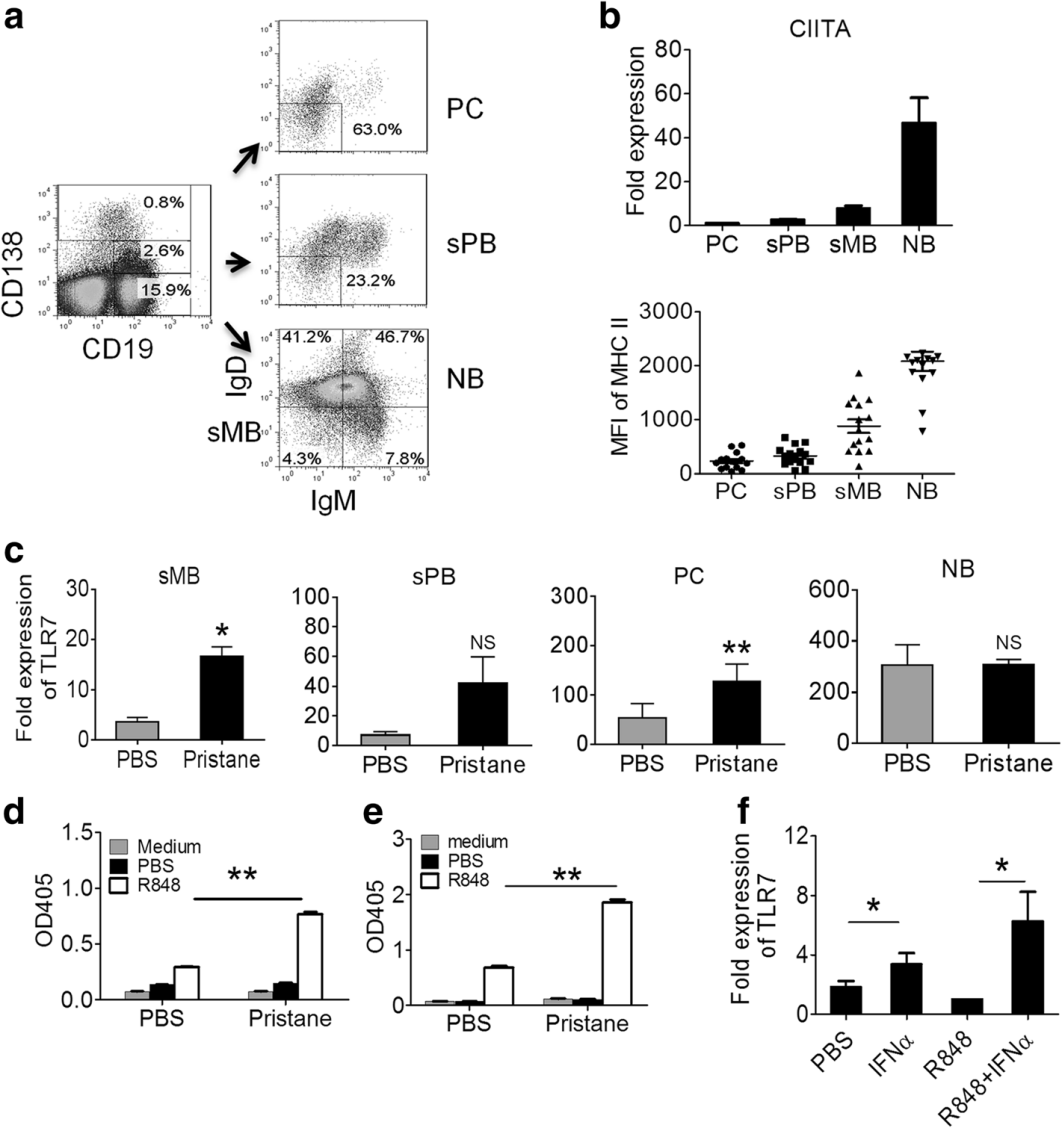
Increased *Tlr7* expression in B cells from pristane-treated mice. Spleen cells from untreated mice were stained with anti-CD19, CD138, IgM, and IgD antibodies and four B cell subsets were identified by flow cytometry and sorted. **a** Gating strategy for isolating naïve B cells (CD19^+^CD138^−^IgM^+^IgD^+^, *NB*), switched memory B cells: CD19^+^CD138^−^IgM^−^IgD^−^, *sMB*), switched plasmablasts (CD19^+^CD138^+^IgM^−^IgD^−^, *sPB*) and plasma cells (CD19^+/−^CD138^++^IgM^−^IgD^−^, *PC*). **b** *Top*, *CIITA* gene expression (Q-PCR) (compared to 18S rRNA) in PC, sPB, sMB, and NB subsets. *Bottom*, spleen cells were stained with anti-CD19, CD138, IgM, IgD, and major histocompatibility complex (*MHC*) II and analyzed by flow cytometry. Mean fluorescence intensity (*MFI*) of MHC II staining in these four B cell subsets is indicated. **c** *Tlr7* gene expression (compared to 18S) in purified sMB, sPB, PC, and NB subsets from pristane-treated and PBS-treated mice. **d** IgG production stimulated by R848 in sPB + PC isolated from pristane or PBS-treated mice and cultured with R848 or PBS. IgG secretion in culture supernatants was measured at 10 days (ELISA). **e** IgG production stimulated by R848 in sMB. **f** *Tlr7* gene expression (Q-PCR) (compared to 18S) in magnetic bead-purified splenic CD19^+^ B cells from PBS-treated mice stimulated by PBS, interferon *(IFN*)α (80U/ml), R848 (1 μg/ml) or IFNα (80U/ml) plus R848 (1 μg/ml) for 6 h: *p <0.05, **p <0.01, Mann–Whitney test

To further investigate the basis for the increased ability of total B cells from pristane-treated mice to produce IgG when stimulated with TLR7 ligands despite comparable *Tlr7* expression (Fig. 1), we asked whether there were differences in *Tlr7* expression or signaling in B cell subsets from pristane-treated vs. PBS-treated mice. *Tlr7* expression was quantified in highly purified B cell subsets from BALB/c mice treated with pristane or PBS. NB (CD19^+^CD138^−^IgM^+^IgD^+^), sMB (CD19^+^CD138^−^IgM^−^IgD^−^), sPB (CD19^+^CD138^+^IgM^−^IgD^−^), and PC (CD19^+/−^CD138^++^IgM^−^IgD^−^) were obtained by flow sorting (Fig. 3a). The sorted PC population also contained some cells that were CD138^++^IgM^−^IgD^+^. As intracellular major histocompatibility complex (MHC) class II molecules promote TLR signaling in myeloid cells by acting as an adapter protein that maintains Btk activation [23], we examined the expression of CIITA, a key transactivator of MHC II expression, in the B cell subsets. In untreated mice, splenic NB expressed the highest levels of CIITA, followed by sMB, sPB and PC (Fig. 3b, top). The same pattern was seen in pristane-treated mice (data not shown). As expected, CIITA expression decreased during terminal differentiation to sPB and PC, confirming the efficacy of the purification scheme. Consistent with CIITA expression, surface MHC II expression also gradually decreased during B cell maturation (Fig. 3b, bottom).

Quantification of *Tlr7* expression by Q-PCR revealed that TLR7 expression levels were higher in NB than in other B cell subsets. However, expression levels were comparable in PBS-treated vs. pristane-treated NB, whereas *Tlr7* was expressed at higher levels by sMB and PC isolated from the spleens of pristane-treated mice vs. PBS-treated controls (Fig. 3c). There was a similar trend in sPB. Because duplication of the *Tlr7* gene promotes autoantibody production in BXSB male mice [24], we examined the responsiveness of isolated splenic sMB (Fig. 3e) and sPB/PC (Fig. 3d) to R848. Both sMB and sPB/PC from pristane-treated mice produced more IgG upon R848 stimulation than the same B cell subsets from PBS-treated controls. As IFN-I plays a key role in pristane-induced lupus [7], we examined its effects on *Tlr7* expression in B cells. Purified splenic B cells from PBS-treated mice were stimulated with IFNα, R848 or R848 + IFNα for 6 h. IFNα alone significantly increased *Tlr7* expression (Fig. 3f). Although R848 alone did not increase *Tlr7* expression, it acted synergistically with IFNα to further increase *Tlr7* expression. These data suggest that the hyper-responsiveness of pristane-treated B cells to R848 stimulation (Figs. 1e; 3d and e) is a consequence of both the increased number of sMB (Fig. 2a) and increased *Tlr7* expression by this subset (Fig. 3c).

### TLR7 agonist induces B cell proliferation and inhibits cell death

**Fig. 4:**
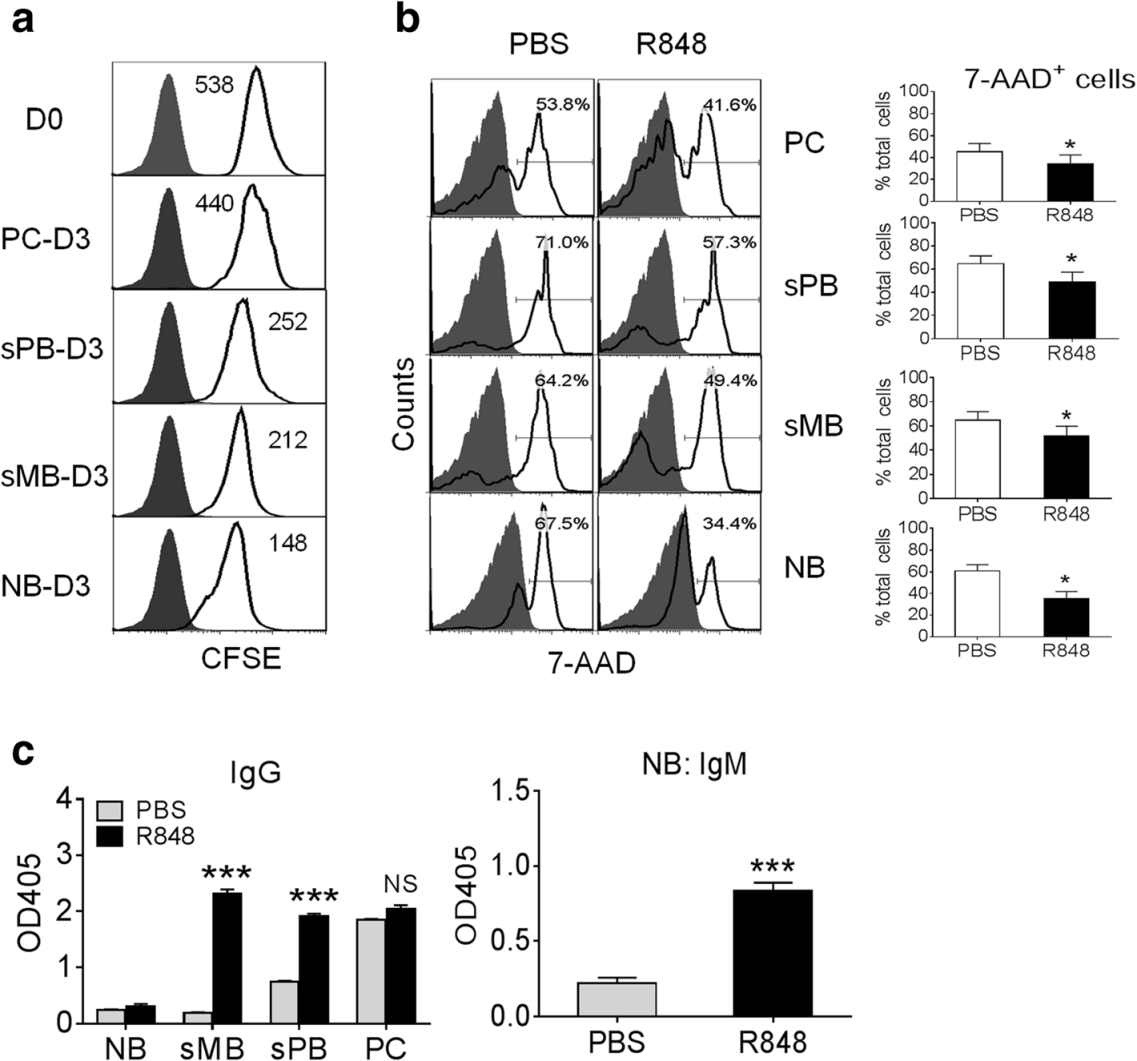
R848-stimulated proliferation, survival, and Ig production by B cell subsets from pristane-treated mice. Splenic naïve B cells (*NB*), switched B memory cells (*sMB*), switched plasmablasts (*sPB*) and plasma cells (*PC*) were flow-sorted from pristane-treated or PBS-treated mice. **a** Sorted B cell subsets from PBS-treated mice were cultured for 3 days with R848. Proliferation was assessed by flow cytometry of carboxyfluorescein diacetate succinimidyl ester (*CFSE*)-loaded cells (*values* are geometric means). **b** Sorted B cell subsets from PBS-treated mice were cultured for 24 h in the presence or absence of R848 and cell death was assessed by 7-AAD staining (flow cytometry). Data are representative of two separate experiments. **c** Sorted B cell subsets from pristane treated mice were stimulated with R848 or PBS for 10 days. IgG and IgM were measured in culture supernatants by ELISA. Data are representative of three separate experiments: *p <0.05; ***p <0.001 (paired Student *t* test)

We next compared the impact of TLR7 ligation on the proliferation and survival of purified B cell subsets (Fig. 4). To assess proliferation, splenic NB, sMB, sPB, and PC from PBS-treated mice were labeled with CFSE, cultured for 72 h with R848, and analyzed by flow cytometry (Fig. 4a). All B cell populations responded, with the greatest proliferation in the NB subset and the least in the PC. The same pattern was seen using B cells from pristane-treated mice, but proliferation was less than in B cells from PBS-treated mice (not shown). As TLR ligands can either induce apoptosis [25] or prevent it [26], we examined the effect of TLR7 ligation on cell death (Fig. 4b). Cells were cultured with R848 for 24 h, stained with 7-AAD and annexin V, and analyzed by flow cytometry. In PBS-treated mice, more than 50 % of B cells in each subset were necrotic (7-AAD^+^) after 24 h in culture. Addition of R848 decreased the percentage of necrotic cells in each B cell subset (Fig. 4b). Among these, NB were the most sensitive to R848, with the percentage of 7-AAD^+^ cells decreasing from 67.5–34.4 %. The survival of sPB, sMB, and to a lesser extent PC, was also improved by culturing with R848. Similarly, culturing B cells with R848 decreased the percentage of apoptotic (annexin-V^+^/7-AAD^−^) cells (not shown).

### TLR7 ligand strongly induces IgG production by sMB from pristane-treated mice

We next examined the effect of R848 treatment on immunoglobulin secretion by B cell subsets from pristane-treated mice. IgG and IgM production by different subsets varied substantially after 10 days of culture with R848 (Fig. 4c). As expected, sPB and PC produced IgG spontaneously but IgG secretion was enhanced in the sPB subset by adding R848 (Fig. 4c left). In contrast, NB and sMB did not secrete IgG spontaneously. However, sMB (but not NB) could be driven by R848 to develop into IgG secreting cells (Fig. 4c left). NB secreted IgM when cultured with R848 but did not switch class (Fig. 4c right). Thus, NB, sMB, and sPB all responded to R848, with the sMB subset showing the most significant increase over baseline IgG secretion. These data suggest that the expanded sMB subset in spleens of pristane-treated mice (Fig. 2a, right) may have an enhanced capacity to undergo R848-mediated differentiation into antibody secreting cells as a consequence of its increased *Tlr7* expression (Fig. 3c).

### Terminally differentiated PCs are unresponsive to TLR7 agonist

PC from pristane-treated mice had increased *Tlr7* expression (Fig. 3c), but did not substantially increase IgG secretion after R848 treatment (Fig. 4c). To further assess whether fully mature PCs respond to TLR7 ligands, we examined the murine plasma cell lines SP2/0 (non-secreting PC) and 2.73 (anti-RNP secreting PC) (Additional file 1: Figure S1). Both cell lines expressed a variety of TLRs, including *Tlr4*, *Tlr7*, and *Tlr9* (Additional file 1: Figure S1A). During terminal B cell differentiation, PCs lose CD19, B220 and MHC II, and gain expression of CD138 [27]. As shown in Additional file 1: Figure S1B, SP2/0 and 2.73 were CD138^+^CD19^−^B220^−^I-A/I-E^+/−^, consistent with a mature PC phenotype. The 2.73 hybridoma and five additional hybridomas of various specificities were cultured with R848, LPS, or CpG1826 for 3 days, and IgG in culture supernatant was quantified by ELISA. As shown in Additional file 1: Figure S1C, TLR4, 7, and 9 agonists had little or no effect on IgG production. There also was little effect on proliferation (Additional file 1: Figure S1D). Thus, although these terminally differentiated PC lines expressed *Tlr4*, *Tlr7*, and *Tlr9*, they did not increase their immunoglobulin secretion or proliferation rate in response to TLR4, 7, or 9 agonists.

### R848 stimulates anti-RNP autoantibody production

**Fig. 5:**
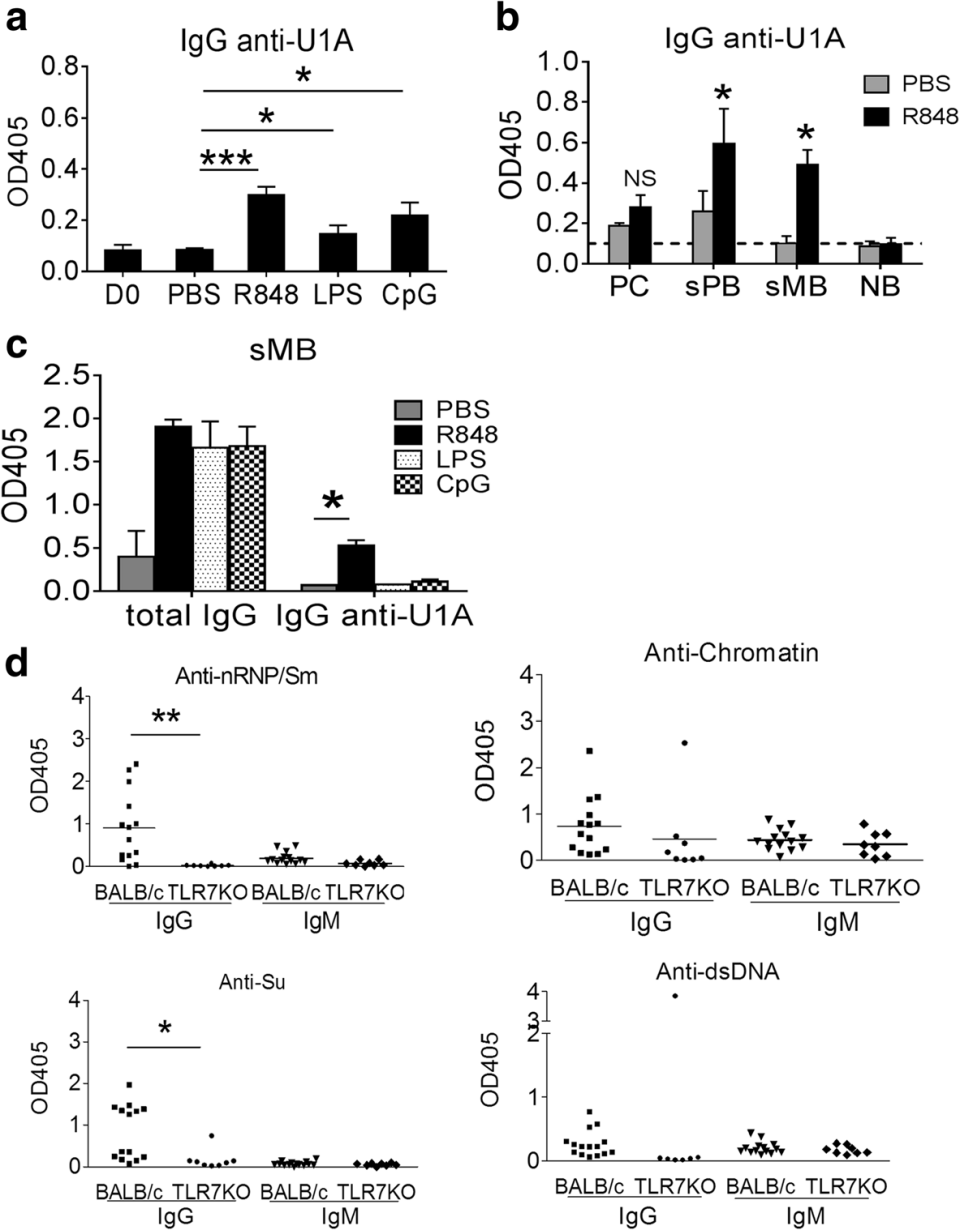
R848 preferentially stimulates anti-U1A autoantibody production by splenic B cells from pristane-treated mice. **a** Purified (magnetic beads) splenic CD19^+^ B from pristane-treated mice were stimulated with toll-like receptor (*TLR*) agonists (R848, *LPS*, or CpG1826) for 10 days. IgG anti-U1A autoantibodies were measured in culture supernatants (ELISA). **b** Flow-sorted plasma cells (*PC*), switched plasmablasts (*PB*), switched memory B cells (*MB*), and naïve B cells (*NB*) were stimulated with PBS or R848 for 10 days, and IgG anti-U1A was measured (ELISA). **c** Flow-sorted splenic sMB cells were stimulated with TLR ligands for 10 days, and total IgG and IgG anti-U1A levels were measured in culture supernatants (ELISA). Data are representative of three experiments. **d** BALB/c and BALB/c TLR7−/− mice were treated with pristane and serum IgG and IgM autoantibody levels were determined (ELISA) 30 weeks later (1:40 serum dilution): *p <0.05, **p <0.01; ***p <0.001 (paired Student *t* test)

TLR7 plays a key role in generating murine autoantibody responses against ribonucleoproteins [6, 10, 20]. However, it is unclear whether TLR7 is involved in the initial activation of anti-RNP/Sm reactive NB to become sMB, the terminal differentiation of sMB into PB/PC, or both. Co-culture with apoptotic cells, containing natural TLR7/9 ligands, enhanced IgG secretion by total B cells from pristane-treated, but not control, mice (Fig. 1b) and B cells from pristane-treated mice were hypersensitive to R848 (Fig. 1e). We examined whether this also was true of autoantibody-producing B cells. Anti-U1A autoantibodies were measured in the culture supernatants of NB, sMB, sPB, and PC sorted from spleen cells of pristane-treated, serum anti-U1A^+^ mice. Total splenic CD19^+^ B cells produced IgG anti-U1A autoantibodies after 10 days culture with R848, LPS, or CpG1826. They responded more strongly to R848 than LPS or CpG1826 (Fig. 5a). IgG anti-U1A autoantibodies were produced spontaneously by sorted PC and PB, and autoantibody production was enhanced (substantially in the MB and PB subsets, with a similar trend in the PC subset) by culturing with R848 (Fig. 5b). The NB subset did not produce IgG anti-U1A after treatment with R848. In the sMB subset, total IgG production was enhanced considerably by R848 as well as LPS and CpG1826 (Fig. 5c). In contrast, IgG anti-U1A autoantibody production by this subset was enhanced more strongly by R848 than by LPS or CpG1826 treatment.

The possibility that pristane treatment might promote production of IgM anti-U1A autoantibodies also was of interest. However, serum levels of IgM anti-nRNP/Sm (which includes anti-U1A), and anti-Su, anti-chromatin, and anti-dsDNA, all were negligible in pristane-treated mice (Fig. 5d). Thus, it proved difficult technically to evaluate IgM autoantibody production from NB in culture.

### Role of R848-stimulated B cell signaling pathways in IgG production

**Fig. 6:**
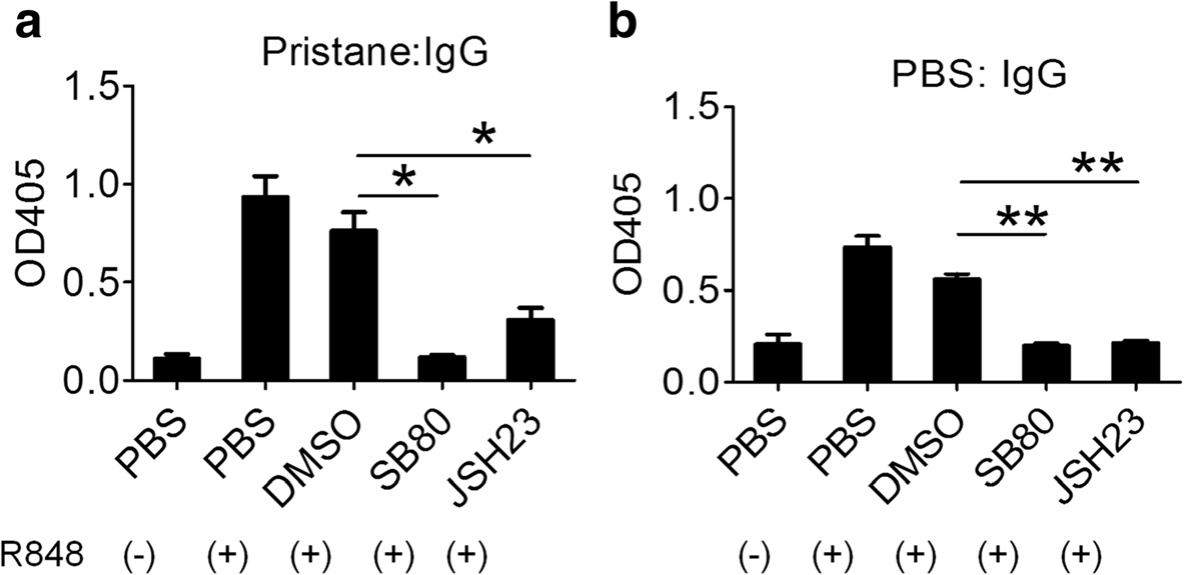
p38 mitogen-activated protein kinase (MAPK) and NF-kB pathways in R848 stimulated IgG production. Purified (magnetic beads) splenic CD19^+^ B cells from pristane-treated mice (**a**) and PBS-treated mice (**b**) were stimulated with R848 alone or with R848 plus p38 MAPK inhibitor SB203580 (SB80) (10 μM) or NF-κB classical pathway inhibitor JSH23 (5 μM) for 10 days. IgG in supernatants was measured with ELISA: *p <0.05; **p <0.01 (paired *t* test) vs. dimethyl sulfoxide (*DMSO*)/R848 control

Upon TLR7 ligation, several signaling pathways are activated via MyD88, including the IRF5/7, NF-κB, p38-MAPK, and PI3K/AKT/mTOR pathways [28, 29]. To investigate the role of NF-κB and p38-MAPK, we stimulated B cells with R848 in the presence or absence of JSH23 (a selective NF-κB classical pathway inhibitor) or SB203580 (a selective p38 MAPK inhibitor). Both JSH23 and SB203580 decreased R848-stimulated IgG production by B cells from pristane-treated (Fig. 6a) and untreated (Fig. 6b) mice, suggesting that both the classical NF-κB and p38-MAPK pathways are involved in TLR7-stimulated IgG production in murine B cells.

## Discussion

Patients with SLE can produce extraordinary levels (titers ≥1:10^6^ or more) of IgG autoantibodies against certain RNA-protein autoantigens, such as U1 snRNPs [1]. Once present, they usually are maintained at high levels for life, consistent with serological memory mediated by long-lived PC, MB, or both. We recently identified sMB-like cells and PC specific for U1A, a component of the U1 snRNP, in BM and spleen of mice with pristane-induced lupus [15]. We examined the role of TLR7 in generating anti-U1A PB and PC from CD19^+^CD138^−^IgD^−^IgM^−^ switched memory-like B cells in pristane-induced lupus. MB from pristane-treated mice produced more IgG than control MB when stimulated with the synthetic TLR7 ligand R848 and expressed higher levels of *Tlr7*, potentially explaining their hyper-responsiveness to R848. Although MB from both untreated and pristane-treated mice produced IgG when stimulated in vitro with R848 (possibly along with antigen released from dying cells in culture), only those from pristane-treated mice produced IgG when co-cultured with apoptotic cells. TLR7 ligand treatment also induced IgG anti-U1A autoantibody production, suggesting that endogenous TLR7 ligands might help to maintain autoantibody production. The potential role of endogenous TLR ligands also is supported by the fact that autoantibodies develop in germ-free mice[30] and are absent in Fas-deficient mice[31]. R848-driven and apoptotic cell-driven IgG production was blocked by an inhibitory oligodeoxyribonucleotide specific for TLR7 (Fig. 1c), and IgG production was decreased in TLR7−/− vs. wild type B cells after stimulation with apoptotic cells (Fig. 1d), consistent with the role of TLR7 ligands in promoting terminal B cell differentiation. JSH23, an inhibitor of the canonical NFκB pathway, and the p38 MAPK inhibitor SB203580 also inhibited R848-driven IgG production. These studies suggest that increased *Tlr7* expression by sMB from pristane-treated mice allows these cells to respond more vigorously to weak TLR7 signals, helping to maintain autoantibody production.

### Production of anti-U1A autoantibodies by PC/PB and memory-like B cells

Serological memory is mediated by long-lived PC as well as MB, which upon antigenic challenge develop into PC [32]. The importance of TLR signaling in generating T cell-dependent, antigen-specific antibody responses is controversial [33, 34], as is the suggestion that serological memory is maintained by polyclonal B cell intrinsic, TLR-mediated, activation of post-germinal center MB [11, 35, 36]. The requirement for TLR signaling may differ depending on the nature of the antigen [11]. In particular, although dendritic cell, but not B cell, MyD88 expression is required to enhance antibody responses using a soluble TLR9 ligand as adjuvant, when the TLR9 ligand is incorporated into a virus-like particle it enhances primary T cell-dependent IgG2a/IgG2c production in a B cell intrinsic, MyD88/TLR9-dependent, manner [37, 38]. Similarly, TLR7 and MyD88 promote primary IgG2a/IgG2c responses to influenza virus by inducing IFNα production [39].

Cellular RNA-protein complexes, such as U1 snRNPs, resemble virus-like particles. When incorporated into U1 snRNPs, U1 RNA is highly resistant to degradation by ribonuclease (W Reeves, unpublished observation), which may delay endosomal degradation, potentially facilitating its association with TLR7. Like antiviral responses, primary autoantibody responses are abolished in MyD88-deficient or TLR7-deficient mice [4, 20, 40]. However, less is known about the role of TLR7 in driving the production of IgG autoantibodies by sMB. Unlike human B cells, in which a CD27^+^ memory subset (and CD27^−^ memory subsets) can be identified readily by their cell surface phenotype [41], the phenotype of murine sMB is less well characterized [42, 43]. We examined CD19^+^CD138^−^IgM^−^IgD^−^ switched memory-like B cells, which may contain both antigen-experienced sMB and activated IgG-expressing non-memory cells. This population was expanded in pristane-treated mice (Fig. 2a) and expressed lower levels of class II MHC and CIITA than NB but higher than PB (Fig. 3b).

The sMB population did not secrete autoantibodies spontaneously, but upon TLR7 ligand exposure produced IgG anti-U1A (Fig. 5b). In contrast, NB (CD19^+^CD138^−^IgM^+^IgD^+^) did not secrete IgG anti-U1A when activated by R848. The anti-U1A B cells identified here differ from the long-lived, low affinity, anti-Sm-specific, mature follicular B-2 cells identified previously in 2-12H/V_κ_8L chain transgenic mice [44], as they were not anergic, but rather were hyper-responsive to TLR ligands (Fig. 5). Anti-U1A B cells in pristane-treated mice also differ from autoreactive B cells in 2-12H chain transgenic mice, which do not spontaneously secrete anti-Sm autoantibodies and have a developmental block at the transitional B cell stage [45]. Some of these cells escape and proceed to an early pre-PC stage, but are subject to tolerance and do not secrete autoantibodies in non-autoimmune mice [46]. Interestingly, this tolerance checkpoint is defective in MRL/*lpr* mice, possibly due to increased B cell signaling. It is interesting to speculate that enhanced TLR7 signaling in B cells from pristane-treated mice might allow autoreactive B cells to escape this early pre-PC checkpoint. Consistent with that possibility, spleen from pristane-treated mice contained an sPB subset (CD19^+^CD138^+^IgM^−^IgD^−^) that secreted anti-U1A autoantibodies spontaneously but had enhanced autoantibody production in response to R848 (Fig. 5b). In view of the hyper-responsiveness of B cells from pristane-treated mice to synthetic TLR7 ligands and apoptotic cells (Fig. 1b), we hypothesize that autologous TLR7 ligands derived from dead cells accumulating within the tissues may help drive the CD19^+^CD138^−^IgM^−^IgD^−^ (sMB) subset in pristane-treated mice to undergo terminal differentiation in vivo. However, further studies will be necessary to confirm that possibility.

### B cells from pristane-treated mice are hyper-responsive to TLR7 ligands

B cells from pristane-treated mice were hypersensitive to R848-stimulated IgG production (Fig. 1a). Interestingly, apoptotic cells also stimulated IgG production in B cells from pristane-treated, but not PBS-treated mice (Fig. 1b). Potential explanations include 1) increased TLR7 expression by pristane-treated B cells, 2) altered signaling downstream of TLR7, or 3) activation of signaling pathways in pristane-treated B cells that act synergistically with TLR7 signaling to promote B cell differentiation or IgG secretion. *Tlr7* gene dosage strongly affects B cell activation and the development of autoantibodies to RNA-protein complexes [24, 47–49]. In addition increased *Tlr7* gene dosage promotes dendritic cell proliferation and cytokine secretion, further upregulating *Tlr7* expression and serving as a positive feedback loop exacerbating autoimmunity [47].

*TLR7* expression is upregulated in PBMCs and B cells from SLE patients [50]. Although we suspected that pristane treatment might increase B cell *Tlr7* expression, expression was similar in purified total CD19^+^ cells from pristane-treated mice and PBS-treated controls (Fig. 1g). However, *Tlr7* expression was increased in sMB, PC, and possibly sPB from pristane-treated mice, but not in NB (the most abundant splenic B cell subset) (Fig. 3c). Switched MB (Fig. 3e) and to a lesser degree PB/PC (Fig. 3d) from pristane-treated mice produced more R848-stimulated IgG than the same subsets from PBS-treated mice. In contrast, NB did not produce IgG in response to R848 but did make IgM, suggesting that TLR7 ligand stimulation could not drive class switch recombination in NB. Interestingly, although serum from pristane-treated mice contained high levels of IgG autoantibodies, levels of IgM autoantibodies were minimal (Fig. 5d), consistent with the possibility that autoreactivity was acquired through somatic hypermutation occurring either in germinal centers or extrafollicular.

Increased *Tlr7* expression in sMB along with increased numbers of sMB may explain the enhanced R848-stimulated IgG production by B cells from pristane-treated mice (Fig. 1e). As IFN-I stimulates *Tlr7* expression in murine B cells and other cell types [51, 52], the strong IFN-I response in pristane-treated mice [7] may increase *Tlr7* expression in sMB (Fig. 3c). It is unclear why NB from pristane-treated mice did not exhibit increased *Tlr7* expression and it will be of interest in the future to see if the NB subset is deficient in *Ifnar1*/*Ifnar2* expression or expression of components of the downstream signaling pathway.

At present, we cannot exclude the possibility that the hyper-responsiveness of B cells from pristane-treated mice reflects altered signaling downstream of TLR7 or in other signaling pathways. For example, FcγRIIB deficiency enhances BCR/TLR7-stimulated B cell proliferative responses [53] and IFNAR/BCR signaling regulates TLR7 responses via activation of the PI3K/Akt/mammalian target of rapamycin (mTOR) signaling pathways [54]. Also accumulating evidence suggests that miRNAs fine-tune TLR signaling pathways by targeting adaptor molecules and TLRs themselves. In particular, miR3148 is predicted to bind the TLR7 3′ UTR, and a polymorphism (rs3853839) may regulate TLR7 expression in SLE [55]. Finally, we cannot exclude a role of enhanced proliferation and/or prolonged survival of splenic PBs (Fig. 4).

## Conclusions

In summary, a population of B cells with a switched memory-like phenotype is expanded in pristane-treated mice, expresses increased levels of *Tlr7*, and is hyper-responsive to synthetic TLR7 ligands and apoptotic cells. This population contains B cells that do not secrete immunoglobulin under resting conditions, but can be driven by TLR7 ligands to produce IgG anti-U1A (RNP) autoantibodies in an NFκB and p38 MAPK-dependent manner. In view of the marked accumulation of dead cells in lupus bone marrow (16) and other sites, we hypothesize that persistently high levels of anti-RNP/Sm autoantibodies in lupus may be maintained, in part, through the chronic activation of sMB by uncleared apoptotic cells.

## Additional file

Additional file 1: Figure S1. Responsiveness of terminally differentiated plasma cells to R848. **a** *Tlr1-9* expression in 2.73 hybridoma and SP2/0 non-secreting myeloma cells (PCR). **b** Surface staining of 2.73 and SP2/0 for CD138, I-A/I-E, B220 and CD19 (flow cytometry). **c** Six different hybridoma cell lines (1: 2.73; 2:172.4; 3:11B11; 4:Pab101; 5:111; 6:162) were stimulated with toll-like receptor (*TLR*) agonists, R848 (TLR7), lipopolysaccharide (*LPS*) (TLR4), or CpG1826 (TLR9) for 3 days labeled with carboxyfluorescein diacetate succinimidyl ester (*CFSE*) followed by stimulationd for 3 days with R848. Cell proliferation was assessed by measuring CFSE fluorescence (values are geometric means). (TIFF 1085 kb)

## Abbreviations

3′ UTR: three prime untranslated region
anti-Sm/RNP: anti-Smith/ribonucleoprotein
BAFF: B-cell activating factor
BM: bone marrow
CFSE: carboxyfluorescein diacetate succinimidyl ester
DMSO: dimethyl sulfoxide
ELISA: enzyme-linked immunosorbent assay
IFN-I: type I interferon
LPS: lipopolysaccharide
mAbs: monoclonal antibodies
MHC: major histocompatibility complex
miR3148: microRNA3148
MyD88: myeloid differentiation primary response gene 88
NB: naïve B cells
NFκB: nuclear factor kappa-light-chain-enhancer of activated B cells
p38 MAPK: p38 mitogen-activated protein kinase
PBS: phosphate-buffered saline
PC: plasma cells
RPMI: Roswell Park Memorial Institute medium
SLE: systemic lupus erythematosus
sMB: switched memory B cells
snRNP: small nuclear ribonucleoprotein
sPB: switched plasmablasts
TLR7: toll-like receptor 7
TNFα: tumor necrosis factor alpha

## References

[1] Reeves WH, Li Y, Zhuang H, Hochberg MC, Silman AJ, Smolen JS, Weinblatt ME, Weisman MH (2011). Autoantibodies in systemic lupus erythematosus. Rheumatology.

[2] Vollmer J, Tluk S, Schmitz C, Hamm S, Jurk M, Forsbach A (2005). Immune stimulation mediated by autoantigen binding sites within small nuclear RNAs involves Toll-like receptors 7 and 8. J Exp Med.

[3] Kelly KM, Zhuang H, Nacionales DC, Scumpia PO, Lyons R, Akaogi J (2006). “Endogenous adjuvant” activity of the RNA components of lupus autoantigens Sm/RNP and Ro 60. Arthritis Rheum.

[4] Lau CM, Broughton C, Tabor AS, Akira S, Flavell RA, Mamula MJ (2005). RNA-associated autoantigens activate B cells by combined B cell antigen receptor/Toll-like receptor 7 engagement. J Exp Med.

[5] Takeda K, Kaisho T, Akira S (2003). Toll-like receptors. Annu Rev Immunol.

[6] Christensen SR, Shupe J, Nickerson K, Kashgarian M, Flavell RA, Shlomchik MJ (2006). Toll-like receptor 7 and TLR9 dictate autoantibody specificity and have opposing inflammatory and regulatory roles in a murine model of lupus. Immunity.

[7] Reeves WH, Lee PY, Weinstein JS, Satoh M, Lu L (2009). Induction of autoimmunity by pristane and other naturally occurring hydrocarbons. Trends Immunol.

[8] Miettinen M, Sareneva T, Julkunen I, Matikainen S (2001). IFNs activate toll-like receptor gene expression in viral infections. Genes Immun.

[9] Christensen SR, Shlomchik MJ (2007). Regulation of lupus-related autoantibody production and clinical disease by Toll-like receptors. Semin Immunol.

[10] Berland R, Fernandez L, Kari E, Han JH, Lomakin I, Akira S (2006). Toll-like receptor 7-dependent loss of B cell tolerance in pathogenic autoantibody knockin mice. Immunity.

[11] Rawlings DJ, Schwartz MA, Jackson SW, Meyer-Bahlburg A (2012). Integration of B cell responses through Toll-like receptors and antigen receptors. Nat Rev Immunol.

[12] Gururajan M, Jacob J, Pulendran B (2007). Toll-like receptor expression and responsiveness of distinct murine splenic and mucosal B-cell subsets. PLoS One.

[13] Genestier L, Taillardet M, Mondiere P, Gheit H, Bella C, Defrance T (2007). TLR agonists selectively promote terminal plasma cell differentiation of B cell subsets specialized in thymus-independent responses. J Immunol.

[14] Bekeredjian-Ding I, Jego G (2009). Toll-like receptors–sentries in the B-cell response. Immunology.

[15] Weinstein JS, Delano MJ, Xu Y, Kelly-Scumpia KM, Nacionales DC, Li Y (2013). Maintenance of anti-Sm/RNP autoantibody production by plasma cells residing in ectopic lymphoid tissue and bone marrow memory B cells. J Immunol.

[16] Zhuang H, Han S, Xu Y, Li Y, Wang H, Yang LJ (2014). Toll-like receptor 7-stimulated tumor necrosis factor alpha causes bone marrow damage in systemic lupus erythematosus. Arthritis Rheumatol.

[17] Billings PB, Allen RW, Jensen FC, Hoch SO (1982). Anti-RNP monoclonal antibodies derived from a mouse strain with lupus-like autoimmunity. J Immunol.

[18] Shin HM, Kim MH, Kim BH, Jung SH, Kim YS, Park HJ (2004). Inhibitory action of novel aromatic diamine compound on lipopolysaccharide-induced nuclear translocation of NF-kappaB without affecting IkappaB degradation. FEBS Lett.

[19] Cuenda A, Rouse J, Doza YN, Meier R, Cohen P, Gallagher TF (1995). SB 203580 is a specific inhibitor of a MAP kinase homologue which is stimulated by cellular stresses and interleukin-1. FEBS Lett.

[20] Lee PY, Kumagai Y, Li Y, Takeuchi O, Yoshida H, Weinstein J (2008). TLR7-dependent and FcgammaR-independent production of type I interferon in experimental mouse lupus. J Exp Med.

[21] Xu Y, Lee PY, Li Y, Liu C, Zhuang H, Han S (2012). Pleiotropic IFN-dependent and -independent effects of IRF5 on the pathogenesis of experimental lupus. J Immunol.

[22] Fukui R, Saitoh S, Kanno A, Onji M, Shibata T, Ito A (2011). Unc93B1 restricts systemic lethal inflammation by orchestrating Toll-like receptor 7 and 9 trafficking. Immunity.

[23] Liu X, Zhan Z, Li D, Xu L, Ma F, Zhang P (2011). Intracellular MHC class II molecules promote TLR-triggered innate immune responses by maintaining activation of the kinase Btk. Nat Immunol.

[24] Pisitkun P, Deane JA, Difilippantonio MJ, Tarasenko T, Satterthwaite AB, Bolland S (2006). Autoreactive B cell responses to RNA-related antigens due to TLR7 gene duplication. Science.

[25] Sohn KC, Li ZJ, Choi DK, Zhang T, Lim JW, Chang IK (2014). Imiquimod induces apoptosis of squamous cell carcinoma (SCC) cells via regulation of A20. PLoS One.

[26] Francois S, El BJ, Dang PM, Pedruzzi E, Gougerot-Pocidalo MA, Elbim C (2005). Inhibition of neutrophil apoptosis by TLR agonists in whole blood: involvement of the phosphoinositide 3-kinase/Akt and NF-kappaB signaling pathways, leading to increased levels of Mcl-1, A1, and phosphorylated Bad. J Immunol.

[27] Oracki SA, Walker JA, Hibbs ML, Corcoran LM, Tarlinton DM (2010). Plasma cell development and survival. Immunol Rev.

[28] Kawai T, Akira S (2007). TLR signaling. Semin Immunol.

[29] Weichhart T, Saemann MD (2008). The PI3K/Akt/mTOR pathway in innate immune cells: emerging therapeutic applications. Ann Rheum Dis.

[30] Mizutani A, Shaheen VM, Yoshida H, Akaogi J, Kuroda Y, Nacionales DC, Yamasaki Y, Hirakata M, Ono N, Reeves WH, Satoh M (2005). Pristane-induced autoimmunity in germ-free mice. Clin Immunol.

[31] Satoh M, Weintraub JP, Yoshida H, Shaheen VM, Richards HB, Shaw M, Reeves WH (2000). Fas and Fas ligand mutations inhibit autoantibody production in pristane-induced lupus. J Immunol.

[32] Manz RA, Hauser AE, Hiepe F, Radbruch A (2005). Maintenance of serum antibody levels. Annu Rev Immunol.

[33] Pasare C, Medzhitov R (2005). Control of B-cell responses by Toll-like receptors. Nature.

[34] Gavin AL, Hoebe K, Duong B, Ota T, Martin C, Beutler B (2006). Adjuvant-enhanced antibody responses in the absence of toll-like receptor signaling. Science.

[35] Bernasconi NL, Traggiai E, Lanzavecchia A (2002). Maintenance of serological memory by polyclonal activation of human memory B cells. Science.

[36] Meyer-Bahlburg A, Khim S, Rawlings DJ (2007). B cell intrinsic TLR signals amplify but are not required for humoral immunity. J Exp Med.

[37] Hou B, Saudan P, Ott G, Wheeler ML, Ji M, Kuzmich L (2011). Selective utilization of Toll-like receptor and MyD88 signaling in B cells for enhancement of the antiviral germinal center response. Immunity.

[38] Jegerlehner A, Maurer P, Bessa J, Hinton HJ, Kopf M, Bachmann MF (2007). TLR9 signaling in B cells determines class switch recombination to IgG2a. J Immunol.

[39] Heer AK, Shamshiev A, Donda A, Uematsu S, Akira S, Kopf M (2007). TLR signaling fine-tunes anti-influenza B cell responses without regulating effector T cell responses. J Immunol.

[40] Green NM, Marshak-Rothstein A (2011). Toll-like receptor driven B cell activation in the induction of systemic autoimmunity. Semin Immunol.

[41] Sanz I, Wei C, Lee FE, Anolik J (2008). Phenotypic and functional heterogeneity of human memory B cells. Semin Immunol.

[42] Bergmann B, Grimsholm O, Thorarinsdottir K, Ren W, Jirholt P, Gjertsson I (2013). Memory B cells in mouse models. Scand J Immunol.

[43] Tomayko MM, Steinel NC, Anderson SM, Shlomchik MJ (2010). Cutting edge: Hierarchy of maturity of murine memory B cell subsets. J Immunol.

[44] Borrero M, Clarke SH (2002). Low-affinity anti-Smith antigen B cells are regulated by anergy as opposed to developmental arrest or differentiation to B-1. J Immunol.

[45] Santulli-Marotto S, Retter MW, Gee R, Mamula MJ, Clarke SH (1998). Autoreactive B cell regulation: peripheral induction of developmental arrest by lupus-associated autoantigens. Immunity.

[46] Culton DA, O'Conner BP, Conway KL, Diz R, Rutan J, Vilen BJ (2006). Early preplasma cells define a tolerance checkpoint for autoreactive B cells. J Immunol.

[47] Deane JA, Pisitkun P, Barrett RS, Feigenbaum L, Town T, Ward JM (2007). Control of toll-like receptor 7 expression is essential to restrict autoimmunity and dendritic cell proliferation. Immunity.

[48] Hwang SH, Lee H, Yamamoto M, Jones LA, Dayalan J, Hopkins R (2012). B cell TLR7 expression drives anti-RNA autoantibody production and exacerbates disease in systemic lupus erythematosus-prone mice. J Immunol.

[49] Moisini I, Huang W, Bethunaickan R, Sahu R, Ricketts PG, Akerman M (2012). The Yaa locus and IFN-alpha fine-tune germinal center B cell selection in murine systemic lupus erythematosus. J Immunol.

[50] Komatsuda A, Wakui H, Iwamoto K, Ozawa M, Togashi M, Masai R (2008). Up-regulated expression of Toll-like receptors mRNAs in peripheral blood mononuclear cells from patients with systemic lupus erythematosus. Clin Exp Immunol.

[51] Green NM, Laws A, Kiefer K, Busconi L, Kim YM, Brinkmann MM (2009). Murine B cell response to TLR7 ligands depends on an IFN-beta feedback loop. J Immunol.

[52] Siren J, Pirhonen J, Julkunen I, Matikainen S (2005). IFN-alpha regulates TLR-dependent gene expression of IFN-alpha, IFN-beta, IL-28, and IL-29. J Immunol.

[53] Avalos AM, Uccellini MB, Lenert P, Viglianti GA, Marshak-Rothstein A (2010). FcgammaRIIB regulation of BCR/TLR-dependent autoreactive B-cell responses. Eur J Immunol.

[54] Poovassery JS, Bishop GA (2012). Type I IFN receptor and the B cell antigen receptor regulate TLR7 responses via distinct molecular mechanisms. J Immunol.

[55] Deng Y, Zhao J, Sakurai D, Kaufman KM, Edberg JC, Kimberly RP (2013). MicroRNA-3148 modulates allelic expression of toll-like receptor 7 variant associated with systemic lupus erythematosus. PLoS Genet.

