# Supplementary figures and images for: Maintenance of autoantibody production in pristane-induced murine lupus
Source: Arthritis Res Ther. 2015 Dec 30;17:384. doi: 10.1186/s13075-015-0886-9 (PMC4718029; doi:10.1186/s13075-015-0886-9)

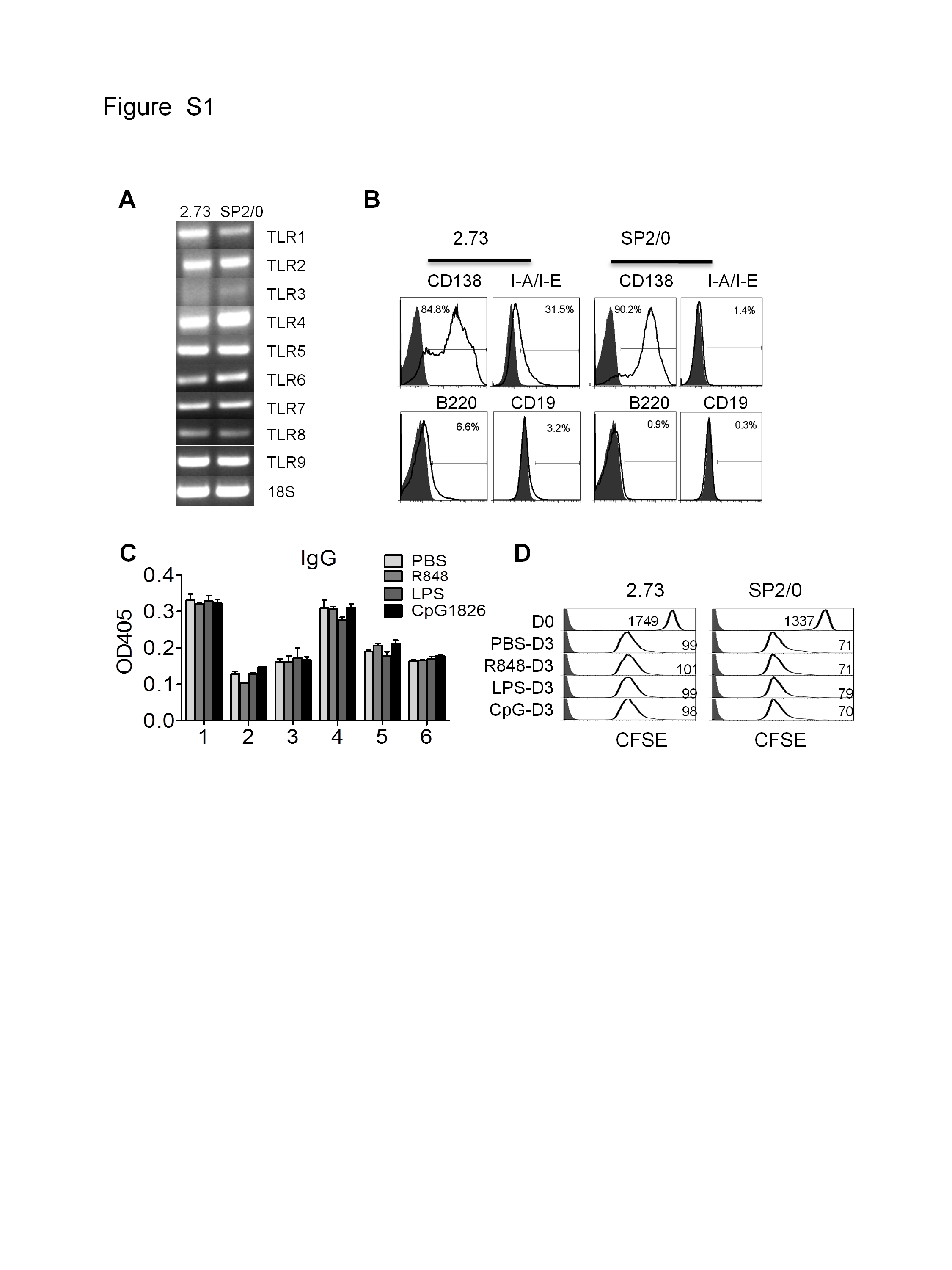

Supplement: Additional file 1: Figure S1. — Responsiveness of terminally differentiated plasma cells to R848. a Tlr1-9 expression in 2.73 hybridoma and SP2/0 non-secreting myeloma cells (PCR). b Surface staining of 2.73 and SP2/0 for CD138, I-A/I-E, B220 and CD19 (flow cytometry). c Six different hybridoma cell lines (1: 2.73; 2:172.4; 3:11B11; 4:Pab101; 5:111; 6:162) were stimulated with toll-like receptor (TLR) agonists, R848 (TLR7), lipopolysaccharide (LPS) (TLR4), or CpG1826 (TLR9) for 3 days labeled with carboxyfluorescein diacetate succinimidyl ester (CFSE) followed by stimulationd for 3 days with R848. Cell proliferation was assessed by measuring CFSE fluorescence (values are geometric means). (TIFF 1085 kb) [file 13075_2015_886_MOESM1_ESM.tiff]
